# Involvement of Hormone- and ROS-Signaling Pathways in the Beneficial Action of Humic Substances on Plants Growing under Normal and Stressing Conditions

**DOI:** 10.1155/2016/3747501

**Published:** 2016-06-05

**Authors:** Andrés Calderín García, Maite Olaetxea, Leandro Azevedo Santos, Verónica Mora, Roberto Baigorri, Marta Fuentes, Angel Maria Zamarreño, Ricardo Luis Louro Berbara, José María Garcia-Mina

**Affiliations:** ^1^Soil Biology Laboratory, Department of Soil, Federal Rural University of Rio de Janeiro (UFRRJ), Rodovia BR 465 km 7, 23890-000 Seropédica, RJ, Brazil; ^2^Department of Environmental Biology, Agricultural Chemistry and Biology Group-CMI Roullier, Faculty of Sciences, University of Navarra, C/Irunlarrea 1, 31008 Pamplona, Spain

## Abstract

The importance of soil humus in soil fertility has been well established many years ago. However, the knowledge about the whole mechanisms by which humic molecules in the rhizosphere improve plant growth remains partial and rather fragmentary. In this review we discuss the relationships between two main signaling pathway families that are affected by humic substances within the plant: one directly related to hormonal action and the other related to reactive oxygen species (ROS). In this sense, our aims are to try the integration of all these events in a more comprehensive model and underline some points in the model that remain unclear and deserve further research.

## 1. Humic Substances and Plant Development 

Numerous studies have reported the beneficial effects of dissolved organic matter (DOM) in both mineral nutrition and development of plants cultivated in many different soil types [1–3]. These effects of DOM are normally related to the presence of a specific fraction of organic molecules known as humic substances (HS). The presence of HS in DOM results from the solubilization and/or runoff of a pool of soil organic matter produced by the transformation of vegetal and animal residues by the action of soil microorganisms and chemical reactions (redox reactions, hydrolysis, and polymerization), both influenced by the environmental conditions (temperature, humidity, soil texture, and pH) [4]. Depending on soil chemical composition and physical features, in soil matrix transformed organic matter is normally associated with minerals, mainly clays and silicates, forming organomineral complexes [4]. These organomineral complexes play a key role in the formation of soil microaggregates, which in turn improve soil porosity, water permeation, and oxygen exchange, thus enhancing soil fertility [2, 4]. In water solution, HS tends to aggregate forming new stable multimolecular systems with specific chemical and biological activities [5, 6]. In this sense, these molecular aggregates can be considered as a family of natural supramolecules or supermolecules [5, 6].

In terms of soil organic matter and more generally due to the complexity and heterogeneity of the humic supramolecules, their definition and classification are rather operational and are based on the different water solubility of HS components as a function of pH and ionic strength [4]: humic acids (HA) (soluble at basic pH but insoluble at acid pH), fulvic acids (FA) (soluble at acid and basic pH), and Humin (insoluble at all pH). Taking into account that HS classification is merely operational, we proposed the introduction in this classification the origin and/or chemical nature of the organic material employed to obtain HA, FA, and Humin [7]. Thus, in this modified classification we have the following. 

*(i) Artificial HS (AHS)*. This indicates organic substances extracted by IHSS-method (IHSS, International Humic Substances Society) from organic materials modified or transformed by using diverse alternative or complementary process different from composting, such as controlled pyrolysis (biochar), chemical oxidation, and anaerobic digestion (digestates). These HS may be named Artificial HS (AHS), and their fractions are as follows: artificial humic acids (AHA) and artificial fulvic acids (AFA). 

*(ii) Fresh HS (FHS)*. This indicates organic substances extracted by IHSS-method from intact, non-biologically or chemically modified, fresh (living) organic materials, such as plant or animal fresh residues (leaves, whole shoot, root, animal or fish flour, wood, and seaweed). These HS may be named Fresh HS (FHS) and their fractions are as follows: fresh humic acids (FHA) and fresh fulvic acids (FFA). 

*(iii) Compost HS (CHS)*. This indicates organic substances extracted by IHSS-method from composted organic materials. These HS may be named Compost HS (CHS) and their fractions are as follows: compost humic acids (CHA) and compost fulvic acids (CFA). 

*(iv) Sedimentary HS (SHS)*. This indicates organic substances extracted by IHSS-method from naturally humified organic matter with sedimentary origin present in terrestrial (soils, coal, leonardite, and peats) and aquatic (lakes, rivers, and sea) environments. These HS may be named Sedimentary HS (SHS), and their fractions are as follows: sedimentary humic acids (SHA) and sedimentary fulvic acids (SFA). 

In principle, only SHS and, by analogy, CHS should properly be considered as HS since they are produced from fresh organic materials and processes both present in natural environments.

A number of studies have reported that HS in solution, mainly SHS and CHS, can affect plant development [1–3, 7]. These effects of HS have normally been differentiated in indirect effects and direct effects [7, 8]. The indirect effects are linked to the action of HS on plant growth by modifying soil or substrate features in the rhizospheric area, principally the pool of potentially bioavailable nutrients and physical texture [7, 8]. The direct effects result from the direct interaction of HS with plant roots or leaves [7, 8]. These effects are reflected in significant changes in metabolic and developmental processes within the plant at transcriptional and posttranscriptional levels [7]. On the other hand, the whole action of HS on plant growth is also influenced by factors that are intrinsic and/or extrinsic to HS in itself. The former is related to HS functional and structural composition and spatial conformation, while the latter is associated with environmental conditions (abiotic and biotic stresses, soil fertility, and crop type), as well as agronomic practices and management (moment of application, doses, and type of application) [7].

In general, the HS concentrations in the rhizosphere that is necessary to affect plant development and mineral nutrition through indirect effects and/or direct effects mediated mechanisms are different to each other [8]. Thus, HS indirect effects can be observed for very low HS concentrations in soil solution (HS < 5 mg L^−1^) since their effects on plant growth are normally linked to their ability to provide complexed micronutrients to plants grown in soils prone to micronutrient deficiency [8–10]. However, consistent HS direct effects are associated with relatively high concentrations of HS in soil solution in the rhizospheric area (HS concentration range: 50–300 mg L^−1^) [8]. Consequently, HS indirect effects mediated actions are probably the main general mechanism involved in the beneficial action of HS in nonirrigated crops growing in dry land types of soils (e.g., Mediterranean climate), while HS direct effects are probably present, along with HS indirect effects, in irrigated crops (pivot, drip, or sprinkle irrigation), where HS are applied through the irrigation system and therefore localized to the root area (greenhouses and open field irrigated crops).

It is well known that HS have beneficial effects on plant development when applied to both the rhizosphere and leaves (foliar sprays) [3, 8]. In this review we deal with the signaling pathways involved in the direct effects on plant development of HS present in the rhizosphere. Taking into account the relevance and novelty of recent results stressing the important role of reactive oxygen species- (ROS-) related signals in HS action on plant roots and plant development under abiotic stresses [11], we also focus our discussion on the crosstalk between the hormonal signaling pathways and ROS-related pathways related to the action of rhizospheric HS.

## 2. Main Signaling Pathways Involved in HS Action in Plants 

Although the mechanism of action of HS has extensively been studied for many years, the knowledge about them is rather fragmentary and it has not been integrated in a comprehensive model yet [7]. In general, all findings reported so far clearly show that HS direct effects mediated action involves several different, but probably interconnected, mechanisms integrated into a complex network of events occurring at both transcriptional and posttranscriptional levels [7].

### 2.1. HS-Mediated Enhancement of Shoot Growth

In the case of rhizospheric HS, direct effects mediated actions have to derive from the interaction of HS with plant roots. Although the possible penetration of some HS fraction into root apoplast and epidermal cell areas has been proposed, results are not very conclusive yet [7]. However, the physical accumulation of HS on root surface has been proved and, therefore, some biological effects on plant development, either on root or on shoot, resulting from it cannot be ruled out [7, 11, 12].

Regarding shoot growth promotion, studies carried out in cucumber with a sedimentary humic acid (SHA) obtained from leonardite reported the important roles of cytokinins (CKs) and mineral nutrient root to shoot translocation in the shoot growth promoting effect of SHA [13]. This effect was, in turn, linked to an increase in plasma membrane (PM) H^+^-ATPase activity and nitrate root to shoot translocation [13, 14]. In line with this potential role of CKs in HS action on plants, further studies on rapeseed showed significant improvements in chloroplast functionality and photosynthesis upon the root application of a SHA obtained from black peat [15]. These physiological effects of SHA were consistent with the upregulation of gene-clusters directly linked to all abovementioned developmental functions such as photosynthesis, CKs signaling perception, and N, S, and C metabolisms [15].

On the other hand, the shoot growth promoting action of SHA in cucumber was dependent on the increase in indoleacetic acid (IAA) and nitric oxide (NO) concentrations in roots caused by SHA root application [14]. Recently, further studies also stressed the crucial role played by an abscisic acid- (ABA-) mediated increase in root hydraulic conductance and aquaporin gene expression in the shoot growth enhancement caused by SHA root application [12]. We have not found any information about the role of a HS-mediated increase in ROS, whether in the root or in the shoot, in the beneficial action of HS on shoot growth. Ongoing studies might throw light on this issue.

**Figure 1:**
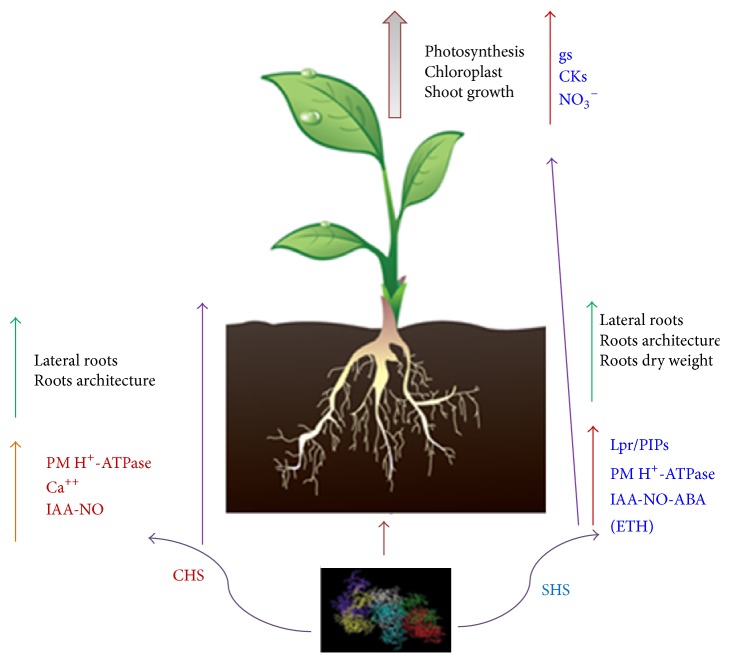
Main hormonal-related signaling pathways affected by humic substances obtained from compost (CHS) or natural sediments (SHS) and some of their biochemical and physiological effects at whole plant level. The metabolic effects of CHS (in red) are associated with stimulation of metabolic pathways belonging to the stimulation of H^+^-ATPase (PM H^+^-ATPase), Ca^++^ transport, and signaling pathways of auxin-nitric oxide (AUX-NO). Therefore, the morphological effects are related to stimulation of lateral roots and improved root architecture. The metabolic effects of SHS (in blue) in plants occur by stimulating the metabolic pathways belonging to the stimulation of H^+^-ATPase enzymes, signaling through pathways of auxin-nitric oxide abscisic acid (Aux-NO-ABA), expression of responsive genes for membrane aquoporins (PIPs) and hydraulic conductivity (Lpr). Therefore, the morphological effects are related to stimulation of lateral root, root architecture improvements, and increased root biomass. These root physiological effects are associated with events in leaf related to stomatal conductance (gs), cytokinins (CKs), and nitrate (NO_3_^−^), finally setting off on effects on leaf growth, photosynthesis, and chloroplasts.

Overall, these results indicate that several interconnected hormone-dependent signaling pathways as well as physiological events are involved in the shoot promotion caused by HA with sedimentary origin (Figure 1).

### 2.2. HS-Mediated Enhancement of Root Growth

Regarding HS direct effects on root development, two main types of phenotypic effects might be distinguished: micromorphological effects (an increase in both absorbent hairs and lateral roots proliferation) and macromorphological effects (an increase in root dry weight, secondary roots number, and primary and secondary roots thickness) [20–23].

Several research groups working with HA obtained from vermicomposts of various vegetal residues (CHA) have reported their ability to promote lateral root proliferation and modify root architecture [21–23]. These studies showed that this effect was associated with an activation of root PM - H^+^-ATPase activity and expressed through auxin (indoleacetic acid, IAA) and probably NO dependent pathways [21–25]. Further studies also suggested that these signaling pathways activated by CHA are expressed at posttranscriptional level through calcium-dependent protein phosphorylation [26]. These effects mediated by hormonal signaling pathways have been shown to be dominated by an upregulation of genes responsive to the synthesis of auxin in both roots (corn and tomato) as whole plant (*Arabidopsis*), as well as other genes encoding metabolic pathways belonging to the taking of nutrients [21–25] (Figure 1).

The effect of CHA has been explained in view of their suprastructural organization. Studies show that the HS may be present and may be released structural fragments and/or molecules to the rhizosphere, with auxin activity and/or structural arrangements similar to auxins [21]. In fact, it is not surprising that composted or vermicomposted fresh vegetal materials have low concentrations of several plant hormones in their molecular systems [21].

The results obtained in studies with HA from sedimentary origin (SHA) also showed the involvement of relevant phytoregulators in SHA-mediated effects on root development [7, 12–15]. The application of SHA to roots of cucumber plants was associated with significant phenotypic effects in roots both at micro- and at macromorphologic scales; these effects were associated with an increase in IAA, ethylene (ETH), NO, and abscisic acid (ABA) concentration in roots. However, further studies showed that the macromorphological effects of SHA could not be explained as a result of its action increasing IAA, ETH, or NO in roots. In consequence, other factors different from IAA, NO, or ETH have to be involved in SHA-mediated increases of root dry weight, secondary roots proliferation, and root thickness [20]. These results are consistent with other results showing that some effects on root architecture of a water soluble fraction of HS obtained from peat were not explained by auxin and/or ETH-dependent pathways in* Arabidopsis* [27] (Figure 1).

Regarding the physical and/or chemical events occurring at root surface as a result of the interaction of SHA with cell walls, the hypotheses proposed for CHA consisting of the presence of hormones and/or auxin-like structural domains in SHA superstructure are not suitable. This is because an extensive analysis of the main plant phytoregulators in SHA by using LC/MS/MS revealed that all these compounds were under detection limits [7, 12–15, 20]. Recent results suggest that the first events caused by SHA at root surface could result from a fouling-mediated transient blockage of cell wall pores, which in turn would be associated with a transient mild water stress, a beneficial stress, “eustress,” that triggers downstream SHA-mediated effects on hormonal signaling pathways and plant development. This mechanism would be linked to the supramolecular conformation of SHA [12].

**Figure 2:**
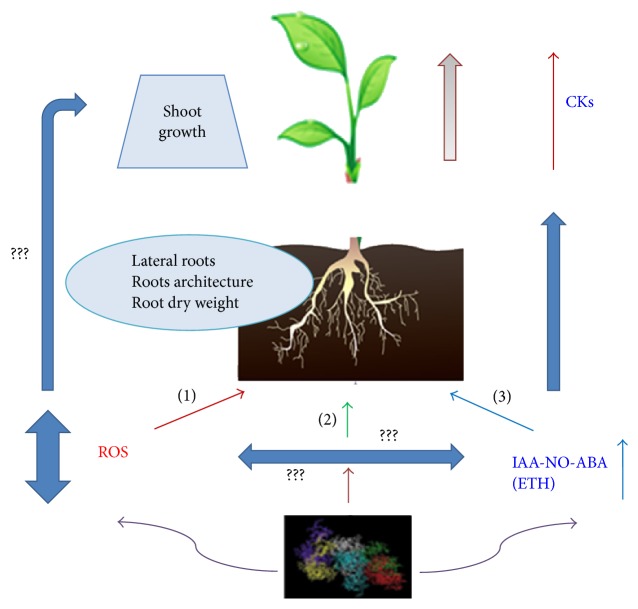
Hypothetical mechanism integrating hormonal-dependent and ROS-dependent signaling pathways in the whole action of humic substances on plant root and shoot. (1) ROS-dependent pathway, (2) hormonal ROS crosstalk pathway, and (3) hormonal-dependent pathway. ???: the causal relationships between IAA-NO-ABA and ROS are yet unknown.

In this context, recent complementary studies suggest the potential role of a new family of molecules involved in signaling in plants [11, 16, 19, 28]. Several studies have shown the relevant role of ROS as an alternative signaling pathway for the regulation and expression of the effects of CHA on lateral root proliferation in rice [16, 19]. Although these results concerning ROS will be discussed below more in depth in connection with other hormonal regulated pathways affected by CHA, these studies clearly showed that CHA increased ROS production and accumulation in roots as well as the antioxidative enzyme network needed to modulate ROS final concentration [16, 28]. The CHA-mediated balance between ROS production and ROS scavenging seemed to be crucial for the signaling role of ROS in CHA direct effects on lateral root proliferation. Interestingly, the CHA-mediated effect on ROS homeostasis in rice roots was associated with a concomitant increase in root dry weight, an effect that was not explained by the increases of IAA, NO, or ETH mediated by SHA in cucumber [16, 19, 20]. This fact suggests that the whole effects caused by HS on root phenotype, both macro- and micromorphological, may involve two pathways, one hormonal-dependent (micromorphological effects) and the other ROS-dependent (macromorphological) (Figure 2).

The involvement of these two pathways is compatible with the role of Ca^2+^-dependent protein kinase activity and protein phosphorylation as second messengers in the posttranscriptional expression of HS activity [26] (Figure 2).

## 3. Crosstalk between Hormone-Mediated Signaling and ROS-Mediated Signaling Pathways in HS Beneficial Effects on Plant Development

As commented above a number of studies have shown the ability of HS to promote plants shoot and root growths [3]. Studies carried out with HA obtained from vermicomposted materials and sediments reported that these HS's beneficial action in plants was functionally linked to an activation of several, interconnected, hormone-mediated signaling pathways; these effects of HS in roots involving some signaling pathways dependent on the hormonal network formed by IAA(ETH)-NO-ABA have been observed in several plant species and have been confirmed using the reporter gene* DR5:GUS* and the expression of auxin-responsive genes, including* IAA19* in* Arabidopsis* [7, 14, 22, 23, 29–32].

Root morphological auxin-like effects and their relationships with other mechanisms of action were investigated by Schmidt et al. [27], who demonstrated that no morphological changes in secondary roots or root hair defective (*rhd6*) mutant recovery occur in* Arabidopsis* with the application of low-molecular-weight HS. This study also showed that* Arabidopsis* strains transformed with the reporter gene* uidA* (GUS) fused to auxin-responsive promoters (*DR5:uidA* and* BA3:uidA*) showed no change or a low response to HS application, concluding that the effects of low-molecular-weight HS may alter root morphology, by increasing the root surface through auxin-independent signaling pathways. In this line, Mora et al. [20] demonstrated that increases in IAA and ETH levels as well as NO in cucumber plant roots had no decisive role in root macromorphological changes (root growth, secondary root proliferation, and root thickness) upon SHA application. The authors conclude that other effects different from those related to IAA, ETH, or NO could act coordinately or independently in stimulating root growth.

In parallel with these studies, other studies reported metabolic changes of reactive oxygen species (ROS) in rice plants upon vermicompost (VC) HA root application. Studies conducted by García et al. [11, 16, 19] demonstrated that the application of cattle manure VC-extracted HA (CHA) to rice plants stimulates certain oxidative metabolism components. CHA application regulates the activity of the enzymes peroxidase (POX), ascorbate peroxidase (APOX), catalase (CAT), and superoxide dismutase (SOD) differently depending on the concentration and exposure time, and this redox regulation simultaneously modulates leaf and root O_2_^∙−^ levels [16]. Regulation of oxidative metabolism in rice plants was demonstrated by the protective effects of CHA when applied to plants subjected to polyethylene glycol (PEG) induced water stress. The presence of CHA at concentrations of 40 mg (C) L^−1^ increased the levels of H_2_O_2_ and decreased lipid peroxidation, possibly via the transformation and control exercised by POX enzymes [19].

**Figure 3:**
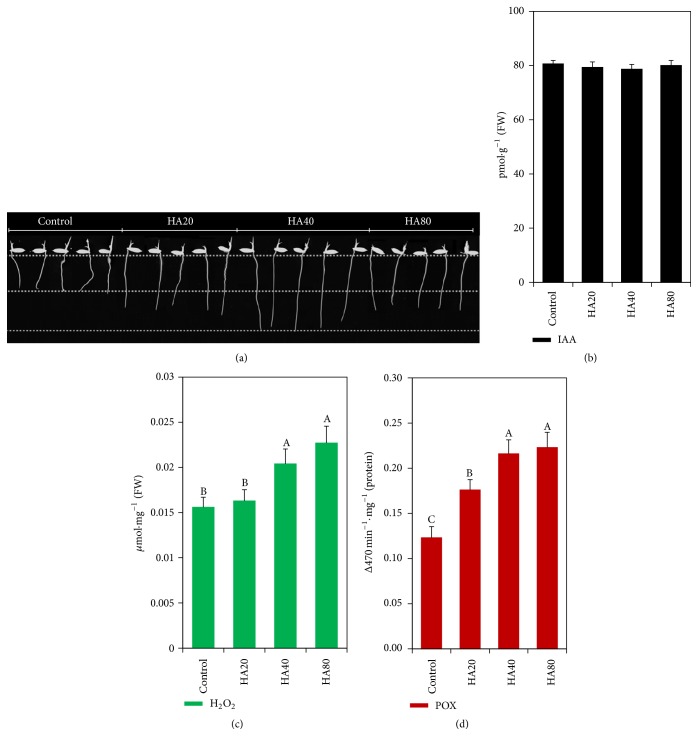
Relationship between rice plant root growth (root length) (a), indole-3-acetic acid (IAA) root levels (b), components of the root redox regulation system (H_2_O_2_ levels) (c), and the relative activity of peroxidases (POX) (d). The stimulation of root growth is dependent on the concentration of humic acid; in this case, 40 mg (C) L^−1^ stimulates the growth of the radicle (a) without the increase of indoleacetic acid concentrations in the roots (b); however, it is possible to observe increase in hydrogen peroxide concentrations (H_2_O_2_) and the peroxidases activity (POX). Different letters represent significant differences between the average values of treatments, as determined by Tukey's test; *p* < 0.05. The error bar represents the mean ± standard error (SE) of three replicates. (Figures were modified from the original papers published at García et al. [16] for better adapting to this review.)

These studies also showed that CHA application promoted lateral root proliferation, in line with a large number of other studies [20, 22, 23, 29]. However, this study also observed an increase in root dry matter associated with no increase in endogenous root IAA levels in response to this application. This result is consistent with results reported by Mora et al. [20] showing that root growth SHA-mediated increase was independent of IAA, NO, and ETH. Conversely, significant increases in root H_2_O_2_ levels and POX enzymatic activity were found in young rice plants (Figure 3).

Taken together, these results show that CHA can, at least in part, regulate the oxidative metabolism associated with root growth. Accordingly, many studies have demonstrated the regulatory role of ROS in many signaling pathways, especially in plants growing under abiotic stresses [33].

**Figure 4:**
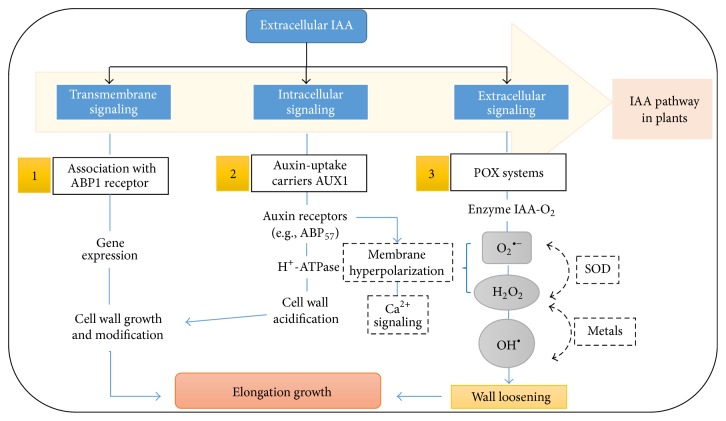
Mechanism of action via auxin signaling pathways compared to the redox mechanism in plant cell growth processes (adapted from [17]).

The results discussed above on the regulation and control of ROS in roots by the action of HA indicate involvement of redox metabolism in the HS mode of action. These forms of action could occur in association (dependent or independent) with known hormonal signaling pathways. In this sense, studies have reported regulatory relationships between hormone and redox metabolism, specifically in signaling pathways related to both auxin metabolism and ROS [34, 35] (Figure 4).

Figure 4 shows three key points for understanding the auxin signaling pathways that may be affected by HS in their action on plant roots. Point 1 in Figure 4 identifies hormone recognition by the ABP1 receptor. Although recent studies showed that the role of this receptor in auxin-mediated recognition must be reevaluated [36], studies have also shown that the auxin signaling pathway is triggered by a perception complex composed of ABP1 and transmembrane kinases (TMK) [36]. However, there is no experimental evidence showing an activation of this ABP1 receptor or ABP1-TMK receptor complex by HS. The lack of evidence of HS binding to this receptor does not invalidate the fact that HS might act by increasing plant auxin synthesis or that auxin-like compounds present in HS, or even auxins themselves released by the structure of HS, might be recognized by these receptors.

Point 2 in Figure 4 shows auxin transport. Cellular influx (AUXIN1/LIKE-AUX1) and efflux (PIN-FORMED (PIN) and P-GLYCOPROTEIN (PGP)) auxin transporters have been identified in the literature, wherein AUX1 is involved in secondary root growth [37]. Although the action of HS on secondary root growth and emission as well as the root stimulation of H^+^-ATPase enzymes has already been demonstrated, studies showing the involvement of auxin transporters in HS-mediated signaling pathways have not been published so far.

Point 3 in Figure 4 relates auxin signaling pathways to redox regulation. Plant oxidative system regulation by HS application was established decades ago by Vaughan et al. [38, 39], who showed that HA and fulvic acid (FA) inhibit the activity of POX in wheat plant roots and stimulate* in vitro *O_2_^∙−^ production [39]. In this line, HA application to maize plants also stimulated CAT activity and ROS production [28]. ROS production and stimulation of oxidative system enzymes have also been shown in rice plants [11]. In this context, few studies have investigated the gene expression codifying this enzymatic system. However, 9% of transcripts produced and stimulated by HA application in* Arabidopsis* have been shown to correspond to stress response metabolism using large-scale gene expression methods (microarray and cDNA-AFLP) [40]. The same response was observed in* Brassica napus*, wherein HA application stimulated 5.8% and 6.6% of stress response-related genes in shoots and roots, respectively [15].

### 3.1. Roles of ROS in Plant Root Growth and the Effects of HS on Plant Development

Most studies have shown a beneficial morphological effect on root system growth, albeit with different metabolic pathways involved in the HS mechanism of action in plants [20, 21]. The involvement of ROS in root cell elongation via Ca^2+^ channel activation is currently known [41]. ROS activation of Ca^2+^ channels is a key step in the regulation of other important processes, including antistress regulation and hormone signaling [42]. ROS produced by NADPH oxidase enzymes create a Ca^2+^gradient in the apical root region, leading to secondary root growth [43]. Stimulation of calcium-dependent protein kinase (CPDK) activity in rice plant roots and increased* OsCPK7* and* OsCPK17* gene expression upon CHA application have been reported [26].

Although studies on the role of ROS as signaling molecules involved in root growth regulation remain ongoing, it has been reported that OH^∙^ species resulting from an increase in apoplast H_2_O_2 _may increase membrane permeability and therefore cellular Ca^2+^ influx [44]. O_2_^∙−^ and H_2_O_2_ are apparently directly related to secondary root growth and emission acting on different regions. Thus, O_2_^∙−^ predominantly accumulates in root elongation regions, whereas H_2_O_2_ accumulates in root differentiation regions [45].

**Figure 5:**
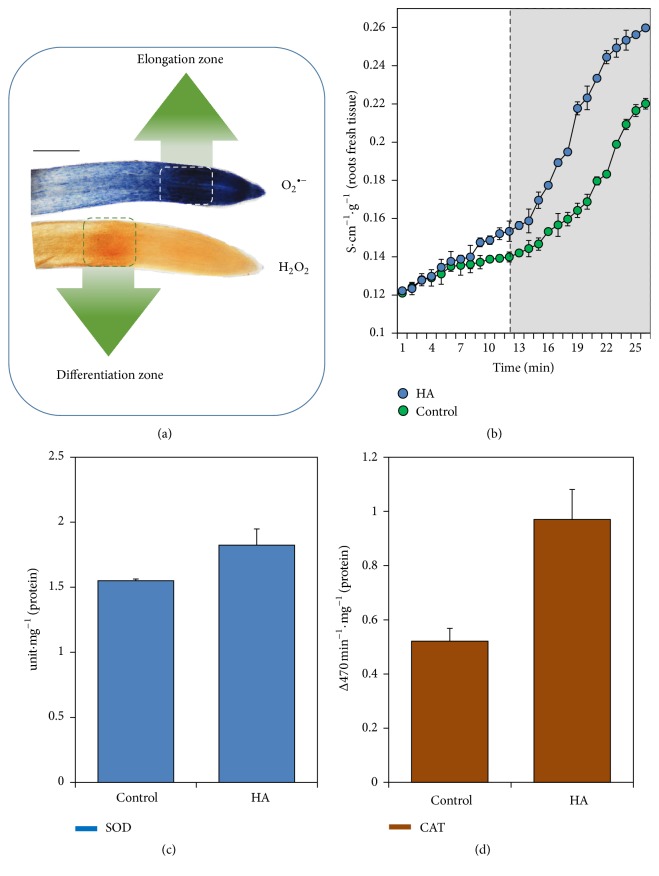
Effect of HA application in roots of rice plant. Histochemistry for determining the location of reactive oxygen species (ROS) (O_2_^∙−^ and H_2_O_2_) (a); membrane integrity (release of electrolytes) (b); superoxide dismutase (SOD) (c); and catalase (CAT) (d) enzymatic activity. The root application HA in plants modulates ROS content in the roots where superoxide anions (O_2_^∙−^) appear to be greater in cell elongation content region, while the H_2_O_2_ content seems to have greater differentiation in region (a). The presence of ROS seems to modify the permeability of the membrane (b) but under control exercised by the antioxidant metabolism (c and d). (These figures were modified from the original papers published at García et al. [18] for better adapting to this review.)

Root CHA application has shown that several components of the redox regulation system are simultaneously stimulated during the root growth promoted by CHA. Root CHA application to rice plants generates increases of ROS located in different root regions depending on the species. The O_2_^∙−^ anions are detected at higher concentrations in the root elongation region of plants, whereas H_2_O_2_ is more concentrated in the root differentiation region (Figure 5(a)).

The CHA was also able to increase membrane permeability, as assessed by the release of electrolytes (Figure 5(b)). Simultaneously, the relative activities of SOD and CAT enzymes directly linked to the transformation and regulation of O_2_^∙−^ and H_2_O_2_ species, respectively, also responded to CHA application (Figures 5(c) and 5(d)).

The roles of ROS in metabolic processes related to plant cell differentiation, specifically those related to redox regulation and cell signaling pathways, are currently an open field of study. However, their involvement in secondary root growth and development is more evident today [46, 47]. Simultaneously, high ROS concentrations in plant tissues, primarily due to the presence of stresses, are known to have toxic effects and may trigger lipid peroxidation and protein denaturation reactions and occasionally lead to cell death [48, 49].

**Figure 6:**
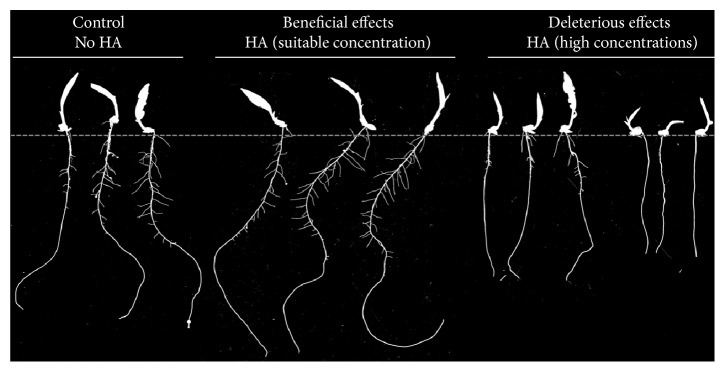
Root system growth and development in* Brachiaria* plants upon application of different concentrations of HA (unpublished data). HA were extracted from vermicompost of cattle manure. Images were recorded using the WinRHIZO software packet (Regent Instruments Inc., Quebec, Canada). Images are the representation of bioactivity experiments (nutrient solution) and reflect the first harvest (eight days after the transplant).

Therefore, the role of ROS as signaling molecules or intermediate species in the HS mode of action in plants seems to respond to a fine adjustment action of redox homeostasis, where the final result is in beneficial effects, including root growth. It is known that this mechanism may be highly dependent on the HS concentration applied to plants. Adverse effects on the root system have been observed in* Brachiaria* upon HA application at high concentrations, and a redox imbalance caused by the high concentrations applied may lead to impaired root growth (Figure 6).

### 3.2. ABA and Aquaporins as Important Factors Involved in the Effects of HS in Plants

ABA is a hormone that plays a key role in plant signaling pathways. ABA regulates and participates in H_2_O_2_ production and Ca^2+^ channel signaling for stomata opening and closing [46, 47]; at the same time, ABA regulates plasma membrane intrinsic protein (PIP) in rice plants under water stress conditions [50]. Others studies have shown that application to rice plants also stimulates the expression of genes of the tonoplast intrinsic aquaporin (TIP) subfamily [51], where the involvement of the TIP subfamily in osmoregulation and water flow through tonoplasts has been demonstrated [52].

Both ABA and TIP-PIP subfamilies are involved in the mechanisms of action of HS in plants. Thus, the root application of SHA to cucumber plants caused a differential increase in ABA levels in roots and shoots [12–14]. Conversely, the application of vermicompost CHA to rice plants subjected to PEG-6000-induced water stress decreased the high levels of ABA in roots under stress [16]. This evidence indicates the involvement of ABA in the hormonal signaling pathways regulated by HA in plants growing under normal and stress environmental conditions. In this line, Olaetxea et al. [12] indicated the crucial role of root ABA and its regulation of root hydraulic conductivity in the shoot growth promoting action of a SHA in cucumber. On the other hand, the relationships between HA mediated effects in plants and root ABA signaling pathways also suggest a potential role of ABA-regulated aquaporins in HS action on plant development. In fact, Olaetxea et al. [12] reported that the ABA-mediated action of SHA was related to the regulation of ABA-dependent PIPs in cucumber roots.

**Figure 7:**
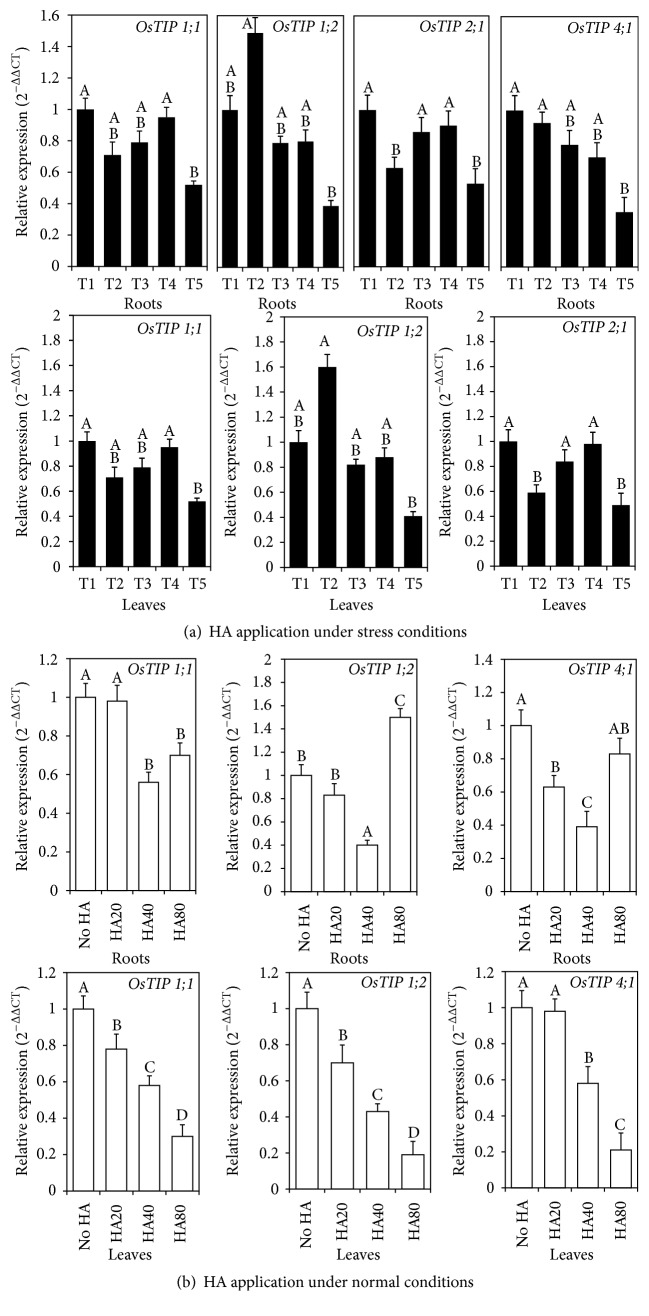
Tonoplast aquaporin gene expression in rice plants subjected to treatments with HA under normal (b) and stress growth conditions (a). (a) (T1) − P–HA: no application of PEG-6000 or HA (control); (T2) + P–HA: PEG-6000 and no HA; (T3) + P + HA20: PEG-6000 and 20 mg (C) L^−1^ HA; (T4) + P + HA40: PEG-6000 and 40 mg (C) L^−1^ HA; (T5) + P + HA80: PEG-6000 and 80 mg (C) L^−1^ HA. (b) HA20 (20 mg (C) L^−1^), HA40 (40 mg (C) L^−1^), and HA80 (80 mg (C) L^−1^) (adapted from [16, 19]). Different letters represent significant differences between the average values of treatments, as determined by Tukey's test; *p* < 0.05. The error bar represents the mean ± standard error (SE) of three replicates.

In this sense, studies performed using CHA applied to roots of rice plants under normal and water stress growth conditions have shown that CHA regulates the gene expression of these isoforms both in leaves and in roots [16, 19] (Figure 7). García et al. [16] also observed that PEG-6000-induced water stress resulted in increased expression of* OsTIP1;2* isoform in rice roots (Figure 7(a)) in line with previous results showing the involvement of* OsTIP1;2* isoform in water flux through vacuoles [50].

Conversely, under normal growth conditions, high CHA concentrations caused increased expression of this isoform, suggesting that CHA application at high concentrations may induce physiological stress [16] (Figure 7(b)). This result confirms that, upon root application, HS concentrations unsuitable for plant growth may trigger physiological processes similar to those developed by plants under stress conditions [53] and can stimulate growth root under normal conditions [54] (Figure 7(b)).

## 4. Concluding Remarks 

Overall, all results discussed above show that the whole mechanism responsible for the beneficial direct effects of HS on plant growth probably involves several complementary and interconnected signaling pathways related to both relevant hormonal networks and secondary messengers such as ROS and Ca^2+^ [7, 11, 21, 54]. In this sense, ROS probably play a pivotal role, either dependent on or independent of hormone signaling, which becomes much more relevant in plants subjected to abiotic stress.

Regarding the relevance of the origin of HS, it was noteworthy that both SHS and CHS presented many common hormone-mediated mechanisms regardless of their potential differences in structure [3, 4]. As for the signaling pathways involved in the mechanism of action of AHS or FHS on plant growth [3], we have not found consistent studies on this subject. However, even though these HS types may be used as biostimulants, their “humic nature” is very questionable. In this framework, studies oriented to better define what we can consider as “humic nature” become of great importance.

Thus, it becomes also clear that much further research is needed in order to integrate all signaling pathways affected by HS with different origins in a more comprehensive, holistic, model.

## Abbreviations

ROS: Reactive oxygen species
DOM: Dissolved organic matter
HS: Humic substances
HA: Humic acids
FA: Fulvic acids
AHS: Artificial humic substances
AHA: Artificial humic acids
AFA: Artificial fulvic acids
FHS: Fresh humic substances
FHA: Fresh humic acids
FFA: Fresh fulvic acids
CHS: Compost humic substances
CHA: Compost humic acids
CFA: Compost fulvic acids
SHS: Sedimentary humic substances
SHA: Sedimentary humic acids
SFA: Sedimentary fulvic acids
CKs: Cytokinins
PM-H^+^-ATPase: Plasma membrane proton pumping
IAA: Indoleacetic acid
NO: Nitrogen oxide
ABA: Abscisic acid
ETH: Ethylene
LC MS/MS: Liquid chromatography-tandem mass spectrometry
DR5:GUS/uida and BA3:uida: Auxin-responsive promoters fused with *β*-glucuronidase reporter gene
IAA19: Indoleacetic acid-induced protein 19
rhd6: Root hair defective 6 (*A. thaliana* mutant)
POX: Peroxidases
APOX: Ascorbate peroxidase
CAT: Catalase
SOD: Superoxide dismutase
ABP1: Auxin binding protein 1
TMK: Transmembrane kinase family
AUXIN1/LIKE-AUX1 (AUX/LAX): Family of major auxin influx carriers
PIN-FORMED (PIN) and P-GLYCOPROTEIN (PGP): Families of proteins are major auxin efflux carriers
cDNA-AFLP: Complementary DNA-amplified fragment length polymorphism
NADPH: Nicotinamide adenine dinucleotide phosphate, reduced form
CPDK: Calcium-dependent protein kinases
PIP: Plasma membrane intrinsic protein
TIP: Tonoplast intrinsic protein.
